# Intestinal necrosis after co‐administration of sodium polystyrene sulfonate and activated charcoal

**DOI:** 10.1002/ccr3.2695

**Published:** 2020-03-12

**Authors:** Namrata Singhania, Raman Al‐Odat, Anil K. Singh, Laith Al‐Rabadi

**Affiliations:** ^1^ Department of Hospital Medicine Mount Carmel East Hospital Columbus Ohio; ^2^ Department of Internal Medicine University of New Mexico Albuquerque New Mexico; ^3^ Department of Internal Medicine Geisinger Community Medical Center Scranton Pennsylvania; ^4^ Department of Nephrology University of Utah Salt Lake City Utah

**Keywords:** activated charcoal, end‐stage renal disease, intentional drug overdose, kayexalate, kidney transplant

## Abstract

Development of acute abdominal pain after Kayexalate and activated charcoal administration should prompt clinician to consider intestinal necrosis. Concomitant use should be avoided to minimize the risk of this devastating but preventable condition.

## Graphical abstract

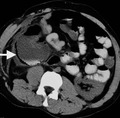

## 1 INTRODUCTION

Sodium polystyrene sulfonate (SPS, Kayexalate), a commonly used resin in the treatment of hyperkalemia, has been implicated in the development of intestinal necrosis. This is a case of a young man who developed this rare complication after administration of activated charcoal‐sorbitol and subsequent use of SPS‐sorbitol.

We report a case of a young man with a history of end‐stage renal disease and kidney transplants who was admitted in the hospital with intentional drug overdose. He received activated charcoal initially for gastric lavage and sodium polystyrene sulfonate (aka Kayexalate) for hyperkalemia and developed colonic necrosis. Our case raises awareness of this rare complication by concomitant use of both activated charcoal and Kayexalate.

## 2 CASE PRESENTATION

### 2.1 History and examination

A 30‐year‐old man presented to the emergency department after ingesting several pills of penicillin, sertraline, and zaleplon with intent to commit suicide. He denied any shortness of breath, fever, abdominal pain, or change in bowel habits at the time of presentation. His past medical history was significant for end‐stage renal disease secondary to streptococcal glomerulonephritis and five kidney transplants secondary to allograft rejection. He is currently on hemodialysis. His family history was negative for any kidney disease or any autoimmune disease. He was nonsmoker. He did not have any history of illicit drug use. On examination, his blood pressure (BP) was 224/150 mm Hg, pulse was 75 per minute, and respiratory rate was 22 per minute. He was slightly drowsy. His lungs were clear to auscultation bilaterally, heart sounds were normal, and abdomen was soft and nontender. He did not have any extremity edema.

### 2.2 Initial work‐up

Laboratory indices showed sodium 135 mmol/L, potassium 5.2 mmol/L, BUN 46 mg/dL, and creatinine 4.5 mg/dL. His electrocardiogram showed normal sinus rhythm. Rest of his blood work was nonsignificant.

### 2.3 Diagnosis and management

He was diagnosed with intentional drug overdose. An emergent gastric lavage was performed, and 50 gm of activated charcoal (AC)‐sorbitol was administered. Nicardipine infusion was started to lower the blood pressure. Twelve hours later, the patient received 15 gm of sodium polystyrene sulfonate (SPS)‐sorbitol orally for potassium of 5.7 mmol/dL. Four hours after the administration of SPS, he started experiencing excruciating right‐sided abdominal pain and had two episodes of nonbloody vomitus. On examination, his bowel sounds were remarkably decreased, and abdomen was distended and diffusely tender. His fecal occult blood test was positive.

### 2.4 Imaging

**Figure 1:**
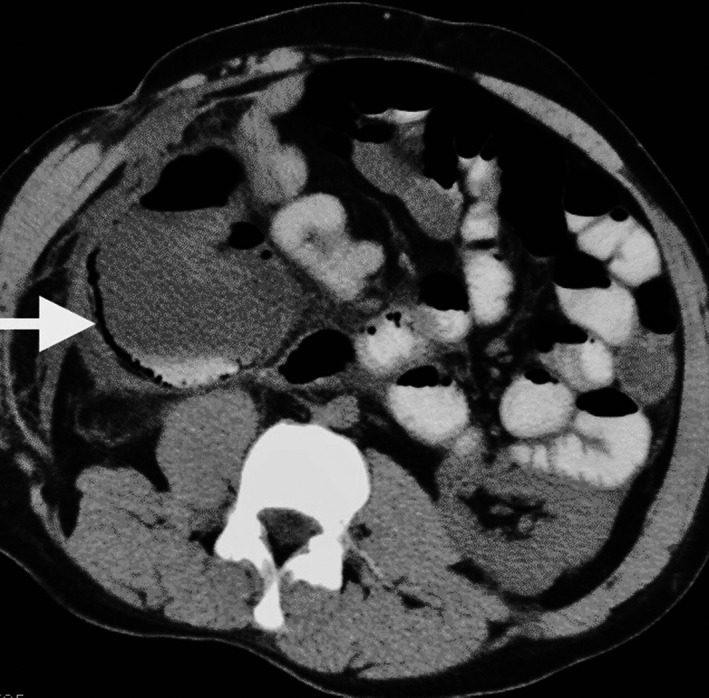
CT abdomen and pelvis showing pneumatosis intestinalis (arrow)

Computed Tomography (CT) abdomen revealed intramural air in the wall of ascending colon (pneumatosis intestinalis) (Figure 1). It also showed distended right colon. Findings were consistent with colonic necrosis.

### 2.5 Treatment and follow‐up

The patient was advised to have surgery, but he opted for conservative management. His condition continued to get worse, and he was transferred to hospice care on patient's request due to poor prognosis from his multiple comorbidities.

## 3 DISCUSSION

SPS (aka Kayexalate) is one of the important drugs given to the patients with hyperkalemia. SPS was initially approved in 1958. Since then, it is being used for hyperkalemia. Earlier, it was used alone but later, sorbitol was added to prevent fecal impaction. Occasionally, articles and case reports have been published regarding its side effects. One of the rare but very important and preventable side effects in patients with uremia or renal transplant or postoperative patients is colonic necrosis. Earlier in 1970s, there were few cases reported with colonic necrosis in patients who received SPS but there were many confounding factors such as uremia, blood volume redistribution, retroperitoneal surgery and immunosuppressive therapy, antibiotic therapy, and irradiation and SPS was not considered causing this.^1^ Later on, in 1987, an experiment was performed in rats which showed that sorbitol component of SPS is the cause.^2^ Although this experiment only studied SPS via enema, later, few more cases were reported of patients with colonic necrosis both with enema as well as oral administration of SPS.^3^ There is also a report showing 33% sorbitol without SPS is not associated with colonic necrosis.^4^ This may suggest that lower concentration of sorbitol decreases the risk of colonic necrosis and can be used safely. Another systematic review reported that this fatal gastrointestinal injury can occur both with and without sorbitol but out of 58 cases, majority (41) preparations contained sorbitol.^5^

The exact mechanism of colonic necrosis is not well known but hypovolemia, hyperreninemia, elevated prostaglandin production, and localized colonic mesenteric vasospasm have all been suggested.^6^ The actual occurrence of colonic necrosis and other related colonic complications is also not well known. Some studies showed incidence of intestinal necrosis was 0.27% and 1.8% in uremic and postoperative patients, respectively.^7^ Most symptoms were seen between 3 hours and 11 days of Kayexalate administration.^7^ Few cases of upper GI tract complications like gastric and duodenal ulcers have also been reported.^6^ Rarely, small bowel necrosis is also seen with SPS administration requiring small bowel resection.^8^ To the best of our knowledge, this case is a first reported case of a patient who received AC and SPS concomitantly. Both had sorbitol in their composition at our hospital. Based on the previously reported studies mentioned above, we believe that our patient was at high risk of intestinal necrosis from sorbitol due to uremia and history of renal transplant.^9^ This case underlines the importance of recognizing a rare, severe, and potentially life‐threatening complication of AC and SPS, and the risk factors associated with it. Concomitant use of AC and SPS should be avoided in these high‐risk patients.

## CONFLICT OF INTEREST

None.

## AUTHOR CONTRIBUTIONS

NS: prepared the manuscript and reviewed the literature. RA, AKS, and LA: reviewed the literature and revised the manuscript.
